# Disseminated pulmonary AL amyloidosis associated with lymphoplasmacytic lymphoma

**DOI:** 10.1002/jha2.193

**Published:** 2021-05-04

**Authors:** Snjezana Janjetovic, Jaroslaw Wojciech Augustyniak, Silvia Darb‐Esfahani, Clemens Baumann, Stephan Fuhrmann

**Affiliations:** ^1^ Department of Hematology and Stem Cell Transplantation Helios Clinic Berlin Buch Berlin Germany; ^2^ Institut for Pathology Spandau Berlin Germany; ^3^ Department of Radiology, Helios Clinic Berlin Buch Berlin Germany

A 76‐year‐old female patient presented with persisting dry cough and dyspnoea. A computed tomography scan of the lungs detected well‐circumscribed consolidation of the middle lobe (Figure 1, panel A, arrows). The laboratory analysis revealed a positive immunofixation for IgM/kappa, elevated serum immunoglobulin (Ig)M (11 g/L), monoclonal protein (1.96 g/L), and free kappa light chains (256 mg/L). Bone marrow biopsy showed lymphoplasmocytoid lymphocytes and plasma cells infiltration. Moreover, monoclonal B cells expressing kappa light chain were identified by flow cytometry (Figure 1, panel B). Chromosome banding analysis showed normal karyotype, while the molecular analysis detected mutation in *MYD88* (L265P) gene. The histopathology of the lung biopsy showed an extensive positivity for congo‐red stain amyloid kappa light chain depositions with typical green birefringence (Figure 1, panels C–E). Thus, the diagnosis of disseminated pulmonary amyloidosis, due to the lymphoplasmacytic lymphoma, was established.

**FIGURE 1:**
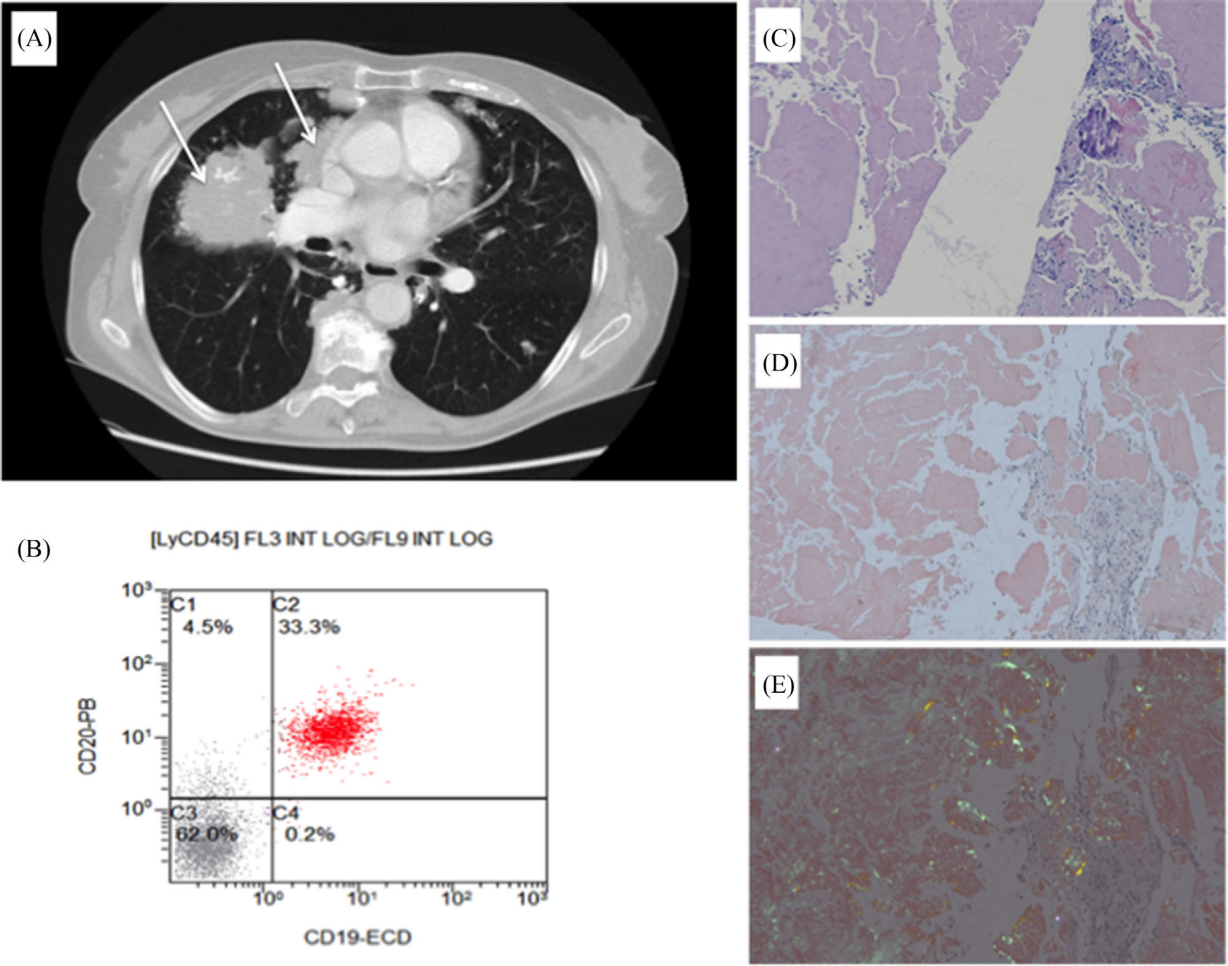
Diagnostic work‐up A: consolidation of the middle lobe by computed tomograpy (arrows). B: monoclonal B cells expressing kappa light chain by flow cytometry. C‐E: histopathology of the lung biopsy showing extensive positivity for congo‐red stain amyloid kappa light chain depositions.

This case represents the disseminated pulmonary amyloidosis as an unusual cause of pulmonary consolidation at presentation of a lymphoplasmacytic lymphoma. Amyloidosis is a rare complication of lymphoplasmacytic lymphomas, and it is found in 3% of all cases and can affect different organs.

## CONFLICT OF INTEREST

S.J.: Financial support of educational meetings by Celgene GmbH, Sobi GmbH, Bristol‐Myers Squibb GmbH, AOP Orphan GmbH, and Amgen GmbH. J.W.A., S.D.E., and C.B.: None. S.F.: Financial support of educational meetings by Amgen GmbH, Celgene GmbH, Roche GmbH, Bristol‐Myers Squibb GmbH, Sanofi GmbH, Gilead GmbH, and Janssen GmbH.

## AUTHOR CONTRIBUTIONS

Snjezana Janjetovic designed and wrote the manuscript; Jaroslaw Wojciech Augustyniak and Silvia Darb‐Esfahani provided the histopathology of the lung biopsy specimen; Clemens Baumann provided computed tomography scan of the lungs; and Stephan Fuhrmann revised the manuscript.

